# Novel assessment of the variation in cervical inter-vertebral motor control in a healthy pain-free population

**DOI:** 10.1038/s41598-021-90306-3

**Published:** 2021-05-24

**Authors:** René Lindstrøm, Alexander Breen, Ning Qu, Alister du Rose, Victoria Blogg Andersen, Alan Breen

**Affiliations:** 1 Department of Health Science and Technology, Aalborg University, Aalborg, Denmark; 2 Centre for Biomechanics Research, AECC University College, Parkwood Rd, Bournemouth, UK; 3 Faculty of Science and Technology, Bournemouth University, Fern Barrow, Poole, UK

**Keywords:** Biological techniques, Biotechnology, Neuroscience, Anatomy, Health care, Medical research, Rheumatology

## Abstract

Spinal control at intervertebral levels is dependent on interactions between the active, passive and neural control elements. However, this has never been quantifiable, and has therefore been outside the reach of clinical assessments and research. This study used fluoroscopy during repeated unconstrained flexion and return neck movements to calculate intersegmental motor control (MC), defined as the difference and variation in repeated continuous angular motion from its average path. The study aimed to determine control values for MC at individual levels and its variability. Twenty male volunteers aged 19–29 received fluoroscopic screening of their cervical spines during 4 repetitions of neutral to full flexion and return motion. Moving vertebral images from C0–C1 to C6–C7 were tracked using cross-correlation codes written in Matlab. MC for each level was defined as the mean of the absolute differences between each repetition’s angular path and their mean and its variability as represented by the SD. 1-way ANOVA and Tukey multiple comparisons were used to identify significant contrasts between levels. The mean MC differences and SDs were highest at C1-2, suggesting that this level has the least control and the most variability. Results at this level alone were highly significant (F-ratio 10.88 and 9.79 *P* < 0.0001). Significant contrasts were only found between C1-C2 and all other levels. The mean MC difference for summed C1-6 levels was 3.4° (0.7–6.1). This study is the first to quantify intervertebral MC in the cervical spine in asymptomatic people. Studies of neck pain patients are now merited.

## Introduction

An improved understanding of the normal spinal control mechanisms at the intervertebral level during functional movements is important for comparison with the symptomatic state when attempting the management of musculoskeletal pain^1^. In Panjabi’s seminal papers^2, 3^, it has been theorised that spinal control is dependent on the interaction between the active, passive and neural control elements. It has also been demonstrated that the cervical spine has an abundance of mechanoreceptors in both its active and passive structures^4^. These act with the vestibular and visual systems to maintain stability during movement^5^. Panjabi proposed that dysfunction in one component is likely to be compensated by adaptations in others and be reflected in parameters reflecting motor control (MC) in the spine. However, due to methodological and technological limitations, there has been little advancement in our understanding of such mechanisms^6^.

There has been considerable focus on MC in neck pain populations, where investigations have addressed various parameters thought to be representative of adaptations in MC. These include; tracking accuracy^7, 8^, smoothness of motion^9^, irregularity of movements^10, 11^ and stiffness^12–14^. All these studies however investigated MC at a global level (i.e. movements of the entire cervical spine), and did not reveal anything about inter-segmental control mechanisms.

There are many reasons why insights into MC at an inter-vertebral level in the cervical spine may be of importance. For example, it is theorised that deep and superficial muscles will contribute differently to motion. Thus, deep locally attaching^15–17^ and proprioceptor-rich muscles^18, 19^ are likely to influence control at an inter-vertebral level, whilst more superficial and globally acting muscles^15^ will act to initiate movement^16^ and effect control over greater ranges of the motion (RoM)^1^. Bogduk and Mercer (2000), found that in non-neck pain populations, RoM is not consistent in any plane^20^, suggesting that strategies vary between movements. Yet whilst RoM may be considered a representation of stiffness, its wide variation in asymptomatic populations reduces its value in clinical assessments. In addition, although changes in global motion have been observed between patients and controls^21, 22^, these changes have not been shown to be significantly different at the inter-vertebral level, including after treatment interventions^23^. This therefore limits their value in terms of understanding pain-related adaptations in MC.

In terms of what these measurements may represent, MC studies that use global measurements (i.e. RoM of entire cervical spine), are most likely to be reflective of superficial muscle activity, while inter-segmental movements better represent the actions of the deeper, segmentally acting muscles^24^. Indeed, it has been shown that abnormal signals from mechanoreceptors in both the active and passive structures can result in altered cervical motion behaviours at specific inter-vertebral levels^25^. Changes in the interactions between motion segments have also been demonstrated in the presence of pain^26^, and may be relevant in clinical interpretations.

Also complicating the interpretation of MC in the cervical spine is the phenomenon of anti-directional intervertebral movement during sagittal bending, where inter-vertebral joints move in the opposite direction to the direction of global motion^27^. This phenomenon is thought to be unique to the cervical spine^28^. This also highlights the necessity for an inter-vertebral assessment of MC there. Thus, there have been calls for measurement techniques that explore continuous motion data at the inter-vertebral level^29, 30^, and *in vivo*^31^.

### Aim

The aim of this study was to investigate the intersegmental MC strategy employed during flexion and return motion in the cervical spine in a healthy population.

The objectives of the study were to determine the MC values for each cervical inter-vertebral motion segment in a healthy control population during a flexion and return task and to determine whether MC varies between inter-vertebral levels.

## Methods

This study used quantitative fluoroscopy (QF) to record continuous inter-vertebral angular movement data from the cervical spine (C1–C7) during repeated sagittal flexion and return. Detailed descriptions of the QF technology are described elsewhere^24^, however, its accuracy for measuring outward and return in plane cervical intervertebral sagittal motion (worst RMS error) has been reported as 0.5° and the worst intra and inter-observer repeatability (highest SEM and lowest ICC) 1.1° and 0.90 respectively^23^.

For this study, the ability of a segment to repeat the same path accurately when the global motion is repeated was taken to be a representation of its MC^32^. Thus, MC for each cervical level was expressed as the average distance between inter-vertebral angular positions at data points averaged over a number of repetitions. The lower the average distance, the greater the MC. The variability of MC can then be considered as the SD of the mean of these distances.

### Participants

Twenty participants were recruited to the study from the undergraduate student population at Aalborg University. All were males between 18 and 29 years old and were free of any neck pain that had caused them to alter their activity for more than one day in the past year. Participants were excluded if they had any significant structural abnormalities in the neck (e.g. fused segments, spondylolisthesis), or if they had received a significant radiation dose (e.g. CT scan or radiotherapy) in the previous year or manipulative treatment to their cervical or thoracic spines in the previous 12 weeks. All participants gave written informed consent before their participation in the study, having been provided with a full written explanation. Participants were paid $22 per hour for their time and the study was conducted in accordance with the Declaration of Helsinki and approved by the Danish Regional Ethics Committee (N20140004).

### Data collection

The age, gender and body mass index of all participants were recorded. All were imaged at the Vejgaard Kiropraktor Klinik in Aalborg by a qualified radiographer using a (Philips BV Libra fluoroscope, 2006, Netherland), using 45 kV, 208 mA, 6.0 ms X-ray pulses. The images were digitized (Honestech VHS to DVD 3.0 SE) and the absorbed radiation dose was recorded for all participants.

Participants were stabilised at the chest and instructed to perform four consecutive neutral to flexion and return neck bending sequences to their maximum tolerable range. This was rehearsed before imaging started. Then, four consecutive video fluoroscopic sequences of the motion were acquired from all participants. Each sequence took approximately 16 s and image sequences included vertebral levels C1–C7. Image sequences were stored securely as MP4 files and given anonymous code names. They were then sent securely in DICOM format to the Centre for Biomechanics Research at the AECC UC in Bournemouth for analysis.

### Data analysis

**Figure 1:**
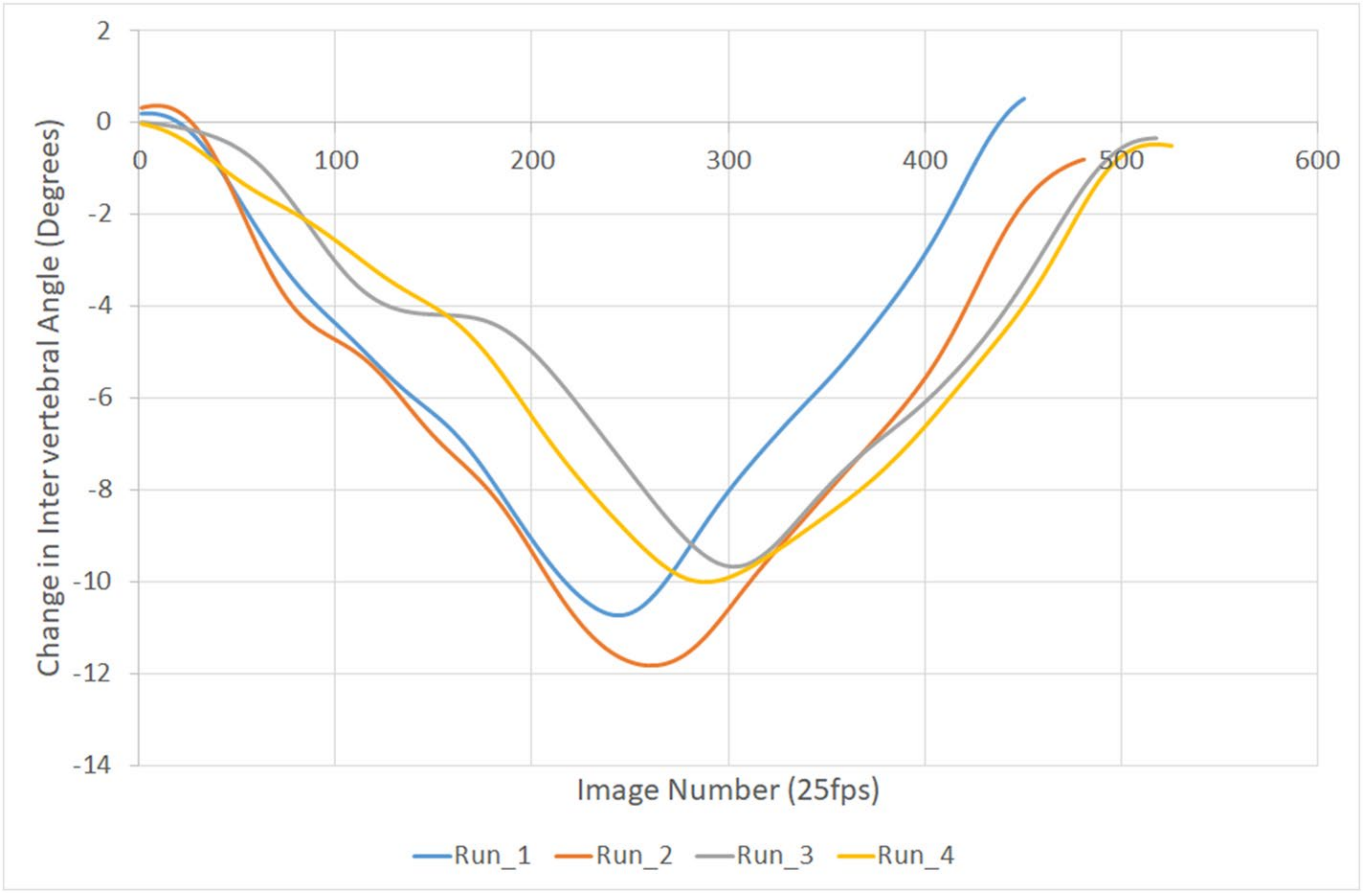
Example of repeated motion paths over time at C3–C4 for one participant.

In Bournemouth, all data were stored in a secure database with image data linked to unique participant identifiers. The vertebral bodies in each sequence were registered and their motion tracked using in-house bespoke image processing codes written in Matlab (The Mathworks, Cambridge). Output from the image processing was in the form of continuous inter-vertebral angles throughout each motion sequence with respect to the starting position of the vertebrae at the beginning of each of the four repetitions of the flexion and return task (Fig. 1).

**Figure 2:**
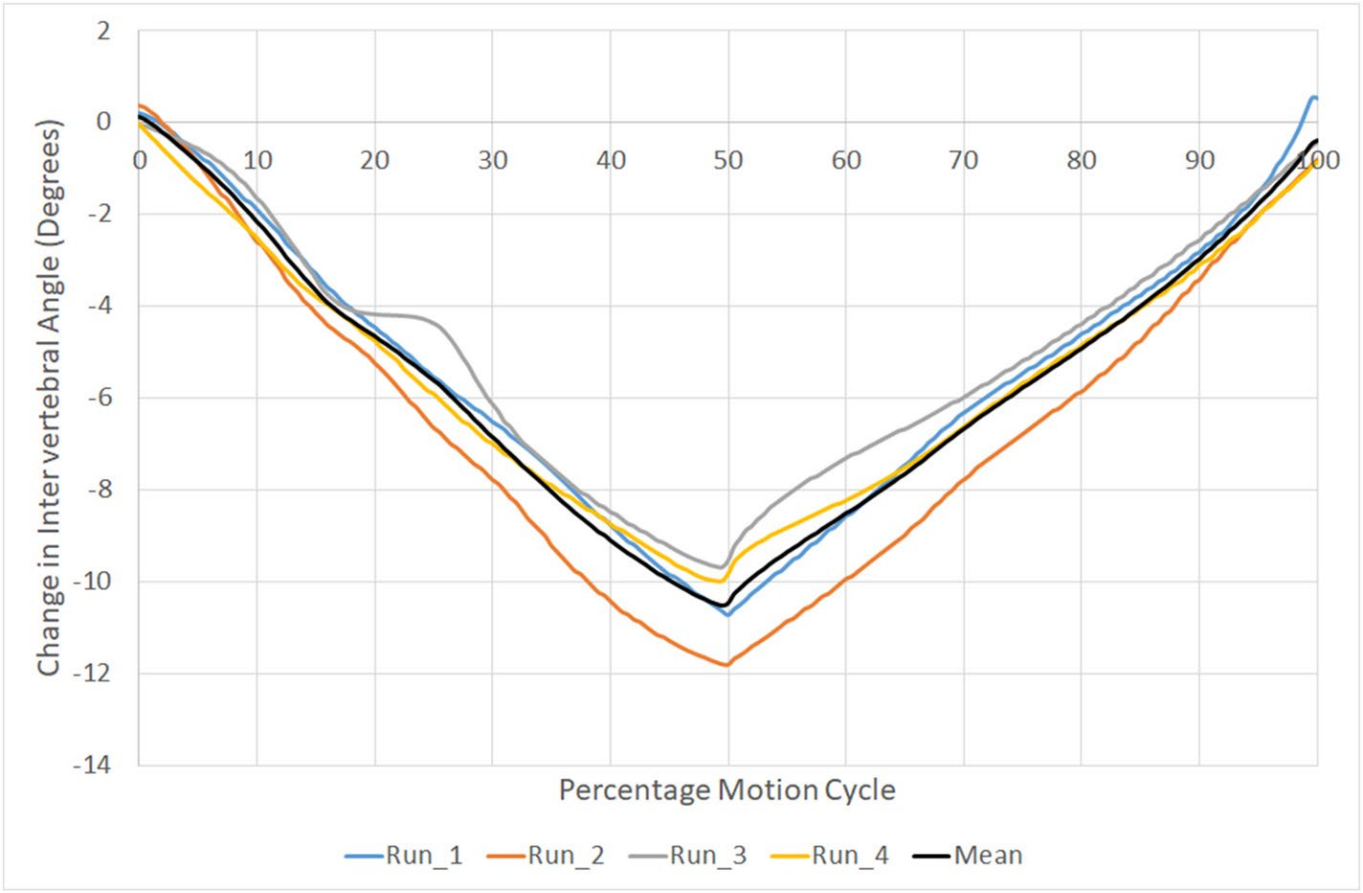
Example of repeated motion paths of C3–C4 for one participant normalised to percentage of the C2–C6 motion cycle.

To compensate for variation in motion onset and speed of participant bending between repetitions, participants’ inter-vertebral angles per repetition were normalised to the motion cycle to allow comparisons between subjects^33^. C2 motion relative to C6 was sampled at 0.5% of each C2–C6 sequence for each participant. In this way, the cervical spine flexion and return per participant was standardized, allowing comparison within and between subjects (Fig. 2). Thus, 0% of the motion cycle was the participant’s position before motion began, 50% was the maximum flexion position of C2–C6 in each repetition and 100% was the return to the neutral position. Segmental kinematics were then interpolated to obtain relative intervertebral motion for every 0.5% increment of C2–C6 spine motion. An example of this normalisation of the same data as presented in Fig. 1 can be seen in Fig. 2.

### Calculation of motor control

**Figure 3:**
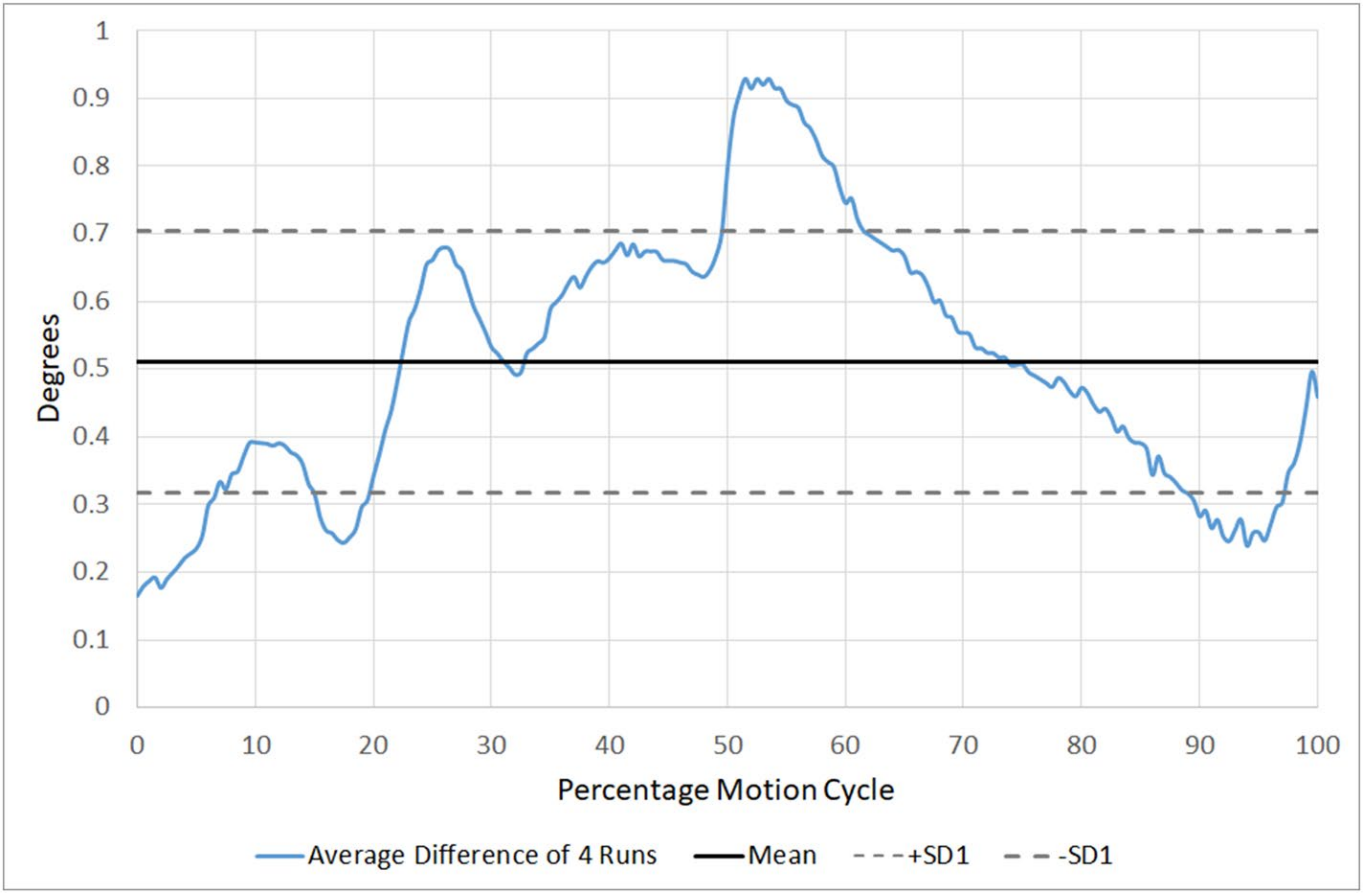
Example of the mean Motor Control and its standard deviation at C3–4 for one participant.

The ability of an intervertebral motion segment to follow the same path was estimated by calculating the absolute difference between each of the 0.5% motion cycle increments for each intervertebral level and their mean (Fig. 2) for four consecutive repetitions of flexion and return. MC of each motion segment was taken to be the mean of these absolute differences and the variation its standard deviation (Fig. 3). Reference ranges were calculated for each motion segment over the population and for the combined motion from C1–C6.

The mean standard deviation of these absolute differences for each motion segment and its composite value (C1–C6) in the population were then calculated as an expression of the normal variation of cervical flexion and return MC.

### Statistical analysis

All data were tested for distribution using the Shapiro–Wilk test and analysed parametrically where normal or near normal distributions across participants were obtained. The mean MC values and their upper and lower reference ranges were then calculated for each level in all participants. One-way ANOVA and Tukey multiple comparisons were performed to identify significant contrasts of the MC between levels.

## Results

Complete data were obtained for all levels from C0 to C7 in 14 participants, from C1 to C7 in 18 participants and from C1 to C6 in all 20 participants. Therefore, to obtain equal datasets for all levels, only the C1–C6 levels were compared for MC. The mean age (SD) of participants was 25 years (3.3, range 18–29), and their mean BMI was 23.6 (2.9). The average radiation dose from the four repetitions was 0.87 Gy cm^2^.

The mean intervertebral neutral-flexion ranges for each level from C0 to C7 are shown in Table 1 and the absolute differences between each of the 4 motion tracks and their means and standard deviations from C1 to C6 in Table 2. The ranges of flexion in Table 1 are comparable to other studies, although their standard deviations are large, indicating why angular range alone is problematical as a measure of the normality of cervical intervertebral motion.

**Table 1:** Intervertebral ranges.

Level	Mean range (°)	SD	Upper ref	Lower ref	n
C0–C1	2.2	2.5	7.0	− 2.7	14
C1–C2	10.6	3.8	18.1	3.2	20
C2–C3	5.5	2.5	10.4	0.6	20
C3–C4	6.7	3.3	13.1	0.2	20
C4–C5	8.0	2.8	13.5	2.4	20
C5–C6	9.1	3.3	15.6	2.7	20
C6–C7	6.7	4.1	14.8	− 1.4	18

**Table 2:** Absolute differences between each of 4 continuous sequential motion tracks and their means (degrees) in population (n = 20).

Level	Mean diff	SD	Upper ref	Lower ref	Mean of SDs	SD of SDs	Upper ref	Lower ref
C1–C2	1.15	0.60	2.34	− 0.03	0.50	0.24	0.96	0.03
C2–C3	0.56	0.25	1.06	0.07	0.22	0.14	0.49	− 0.04
C3–C4	0.55	0.30	1.13	− 0.03	0.25	0.14	0.52	− 0.02
C4–C5	0.58	0.28	1.12	0.04	0.25	0.14	0.52	− 0.02
C5–C6	0.58	0.25	1.08	0.08	0.24	0.11	0.46	0.02
C1–C6*	3.42	1.38	6.13	0.72	1.46	0.59	2.61	0.31

*Sum of Means and SDs.

Both the mean absolute differences and their standard deviations were greatest at C1-2, indicating that there is the least MC at that level (Table 2). 1-way Analysis of Variance revealed differences between levels that were significant both in terms of the differences of intervertebral angles from their means (F-ratio 10.88, *P* < 0.0001) and their standard deviations (F-ratio 9.79, *P* < 0.0001). Tukey multiple comparisons of levels showed significant contrasts between C1 and C2 and all other levels both for differences and standard deviations (*P* < 0.0001). No significant contrasts were found between any other pairs of levels for either differences or standard deviations. The mean difference for the composite value (C1–C6) was 3.4° (reference range 0.7–6.1), which may be taken to represent the composite flexion and return MC of the population.

## Discussion

The study results provide preliminary MC values for each cervical inter-vertebral segment during a flexion and return task in young, healthy male controls, where the average difference between inter-vertebral angular positions over 4 repetitions of a cervical flexion and return task, provides a novel representation of MC. Although it was shown that there is generally consistent control at levels between C2 and C6, there was less consistency of the movements at C1–C2. This performance of pain free movement with greater variation at C1–C2 suggests that the reduced MC at this level is normal.

### What is causing the results/differences between levels?

That C1-2 may be biomechanically distinct from the other cervical segments in terms of its motor control capacity is not unexpected and is likely to reflect changes in afferent input and its unique muscular morphology as well as the absence of an intervertebral disc at that level. It has been suggested by Anderst et al.^31^ that the greatest variation in intervertebral range of motion during flexion tasks also occurs at C1–C2, influenced by region-specific deep localised posterior muscles such as the rectus capitis posterior major and minor, and the obliquus capitis superior. However, recent studies have also demonstrated that the lower cervical spinal levels receive most of the sagittal motion during flexion tasks. In the present study however, missing data due to the image field size prevented analysis of C6-T1^34, 35^.

It has also been suggested that a combination of feedforward and feedback control^32^ acts as a mechanism to modify muscle contractions and influence spinal stiffness and thus, MC. It is apparent from this study however, that MC strategies are intrinsically different in the upper cervical spine compared to the middle, with less variation evident for the latter.

Studies that have investigated cervical muscle activation patterns in neck pain populations provide valuable insight into adaptive MC mechanisms and some common changes in muscle activation have been observed in such patients. In terms of the segmentally acting deep neck flexors, including the longus capitus and the longus colli, inhibition and delayed activity onset and decreased endurance have both been demonstrated in patients^36^, with a concurrent increase in activation of the more globally acting superficial neck flexors^37, 38^. However, whilst changes in muscle activation levels in both patient and pain-free groups will influence cervical movements, it is not believed that the superficial muscles have any direct control over cervical inter-vertebral joints^24^. It is therefore proposed that changes in MC in such symptomatic populations are a result of changes in the level of activity in the surrounding deep musculature and in the proprioceptive capacity of both active and passive restraining structures.

There is disagreement in the literature regarding the changes in mechanisms of control observed in neck pain patients. In terms of stiffness for example, it has been suggested that any increased variability observed in this group would be the result of a decrease in proprioceptive input^39^. Other studies agree, having shown that neck pain patients appear to have an increase in irregular and uncoordinated global movements^40–43^ which are theorised to result from alterations to the sensory motor processing in the neck, but may also be influenced by discomfort avoidance. Such findings have been shown to persist even at long term follow up, suggesting a persisting adaptive MC strategy^42^. In contrast however, others have observed a decrease in variability, a motor control strategy attributed to increased muscle activation of the superficial muscles or via co-contraction of agonist–antagonist muscles stiffening the region. It is likely that such MC strategies are individual-dependent, and that increases and decreases in movement variability are both found in neck pain populations. It should be noted that whist segmental motion distribution may be influenced by voluntary control according to an individual’s movement preferences, this may not be the case with repeated motion. The values for segmental motion provided in this study provide an indication of the baseline from which such variability in symptomatic cohorts could be compared.

### Why are inter-vertebral insights important?

It is also intuitive that the morphology of the cervical spine may influence its movement. A recent study however showed there to be no difference between the cervical lordosis of neck pain patients and non-neck pain controls^44^. In the lumbar spine however, lordosis has been demonstrated to affect motion segment interactions^45^, and is therefore likely to be worthy of further exploration in the cervical spine. Indeed, whilst in cadaveric experiments, it has been shown that motion compensation associated with single level fusions occurs at the segmental levels immediately adjacent to the fused level^31, 46, 47^, recent in vivo studies have shown a co-dependence between motion segments at different levels of the cervical spine^24, 26^ while relationships between levels have often been shown to be distant from each other (e.g. C1–C2 and C5–C6).

### Limitations and further work

A limitation of this study was missing data at C0–C1 and C6–C7, which was due to limitations of the X-ray equipment. A larger sample including females and a wider age range would also be desirable, but has to be balanced with the benefits and risks associated with irradiation of a healthy cohort in an exploratory study. Although a young male student population may bring a measure of homogeneity, future studies could also include information about morphology (e.g. lordosis, disc degeneration) and activity levels. An obvious next step would be to repeat the investigation in neck pain groups, in order to compare with the non-neck pain data. This could also incorporate the concurrent use of electromyography to record muscle activity directly. It would also be of interest to investigate the effect of muscle fatigue on the findings.

The present study provides insight into why C1–2 demonstrates the most variability in a healthy population. This may be because this level has the greatest capacity to adapt, but whether this capacity is increased or diminished in symptomatic subjects must await future studies. The relative prevalence of paradoxical (or anti-directional) motion, which has been shown to be a normal phenomenon in the cervical spine most frequently found at C1–2, in symptomatic and asymptomatic groups, might also become better explained by such studies.

Finally, it would also be of interest to analyse the results when divided into different epochs of the flexion and return phase. Wu et al. (2010) showed that during flexion, the majority of motion takes place in the middle cervical segments during initial flexion and in the lower segments during the latter stages of movement^48^. It would therefore be of interest to see if MC varies between these stages.

## Conclusion

This is the first study to describe intersegmental MC and its variability in the cervical spine using a novel representation based on kinematics. Through the use of QF, it was possible to quantify segmental movement accurately during a cervical flexion task in a group of participants with no neck pain. Data were recorded for cervical levels C1-C6, and notably less control was shown at C1-2 than in the rest of the cervical levels, which demonstrated consistently similar amounts of MC. These parameters provide a segmental reflection of an individual’s cervical control system. Whilst they can initially be used as a limited dataset of healthy controls, a larger and more representative sample will be needed with which to compare motor control in neck pain patients.
